# Deconvolved Fractional Fourier Domain Beamforming for Linear Frequency Modulation Signals

**DOI:** 10.3390/s23073511

**Published:** 2023-03-27

**Authors:** Zhuoran Liu, Quan Tao, Wanzhong Sun, Xiaomei Fu

**Affiliations:** School of Marine Science and Technology, Tianjin University, Tianjin 300072, China

**Keywords:** deconvolved beamforming, fractional Fourier transform, direction of arrival estimation, linear frequency modulation signal

## Abstract

To estimate the direction of arrival (DOA) of a linear frequency modulation (LFM) signal in a low signal-to-noise ratio (SNR) hydroacoustic environment by a small aperture array, a novel deconvolved beamforming method based on fractional Fourier domain delay-and-sum beamforming (FrFB) was proposed. Fractional Fourier transform (FrFT) was used to convert the received signal into the fractional Fourier domain, and delay-and-sum beamforming was subsequently performed. Noise resistance was acquired by focusing the energy of the LFM signal distributed in the time–frequency domain. Then, according to the convolution structure of the FrFB complex output, the influence of the fractional Fourier domain complex beam pattern was removed by deconvolution, and the target spatial distribution was restored. Therefore, an improved spatial resolution of DOA estimation was obtained without increasing the array aperture. The simulation and experimental results show that, with a small aperture array at low SNR, the proposed method possesses higher spatial resolution than FrFB and frequency-domain deconvolved conventional beamforming.

## 1. Introduction

Linear frequency modulation (LFM) signals are widely used in hydroacoustic communication and detection, and direction of arrival (DOA) estimation is important [1,2]. To estimate received signal DOA, beamforming methods represented by conventional beamforming (CBF) [3] and minimum variance distortionless response (MVDR) [4] were proposed. However, those methods cannot directly estimate the wideband signal DOA due to the steering vector being frequency-dependent [5].

An intuitive approach to extend those narrowband beamforming methods to wideband beamforming is dividing the wideband signal into a set of narrowband signals in the frequency domain. Following this idea, two classical frameworks, the incoherent signal-subspace method (ISM) [6] and the coherent signal-subspace method (CSM) [7], were proposed. The ISM framework processes each subband signal separately and obtains a comprehensive result by combining all subband results. The CSM framework aligns each subband datum to a reference frequency by focusing matrices and obtains a result via the focused data. Those two frameworks solve the problem that a single-frequency steering vector cannot perform beamforming on wideband signals but fail to make full use of the time–frequency characteristics of LFM signals [8]. Various time–frequency analysis tools were introduced to utilize the characteristics of LFM signals. Wigner–Ville distribution (WVD)-based methods [9,10] possess a good estimation performance but suffer from the cross terms between multiple targets [11]. Spatial time–frequency distribution (STFD)-based methods [12,13] can directly exploit the nonstationarity information of LFM signals but are highly dependent on time–frequency point selection [12]. Matched filtering (MF)-based methods [14,15] possess a high noise resistance by treating MF as pre-processing or post-processing of beamforming.

The fractional Fourier transform (FrFT) [16,17,18,19] is a generalization of the classical Fourier transform. FrFT can be regarded as the chirp basis decomposition [20], which means it is an extremely effective time–frequency analysis tool for LFM signals. Fractional Fourier domain (FrFD) beamforming methods [8,21,22] can utilize the energy-focusing characteristic of LFM signals by FrFT. The LFM signals are transformed into narrowband signals in the FrFD, and the DOA can be estimated by narrowband beamforming methods. The performance of FrFD beamforming methods is significantly better than that of ISM-based and CSM-based methods, especially at low SNRs [8,23]. Different from the classical time–frequency domain, there are no cross terms in FrFD. The selection of a time–frequency point can be easily achieved by peak search [24]. FrFT has also shown several advantages over MF regarding robustness against distortion [25]. Therefore, beamforming methods based on FrFT are the best in terms of making full use of the time–frequency characteristics of LFM signals. Among all FrFD beamforming methods, fractional Fourier domain delay-and-sum beamforming (FrFB) is one of the most popular methods. FrFB inherits high robustness from CBF but suffers from wide beamwidth, high sidelobes, and limited spatial resolution [8].

Yang proposed deconvolved conventional beamforming (DCBF) to improve the DOA estimation performance of CBF without increasing the array aperture [26]. By regarding the CBF spatial spectrum as the beam pattern convolution with the target spatial distribution, the Richardson–Lucy (R-L) [27] algorithm can be used to restore a high-resolution spatial spectrum. In recent years, DCBF has been widely used for various arrays [28,29,30,31,32,33,34,35,36,37,38]. Deconvolution is also used as postprocessing for other beamforming methods, such as Chebyshev weighting beamforming [39] and frequency-difference beamforming [40], to optimize their spatial spectrum and to improve resolution.

The abovementioned deconvolved beamforming methods all obtain a spatial spectrum with a narrow beamwidth and low sidelobes, which effectively improves the spatial resolution. However, the beam pattern used in deconvolution is a function of frequency, which is applicable to only narrowband signals. A direct wideband deconvolved beamforming method, named ISM-DCBF in this paper, is to divide the LFM signals into several narrowband signals in the frequency domain following the ISM and to use DCBF for each subband signal. In ISM-DCBF, the deconvolution is repeated multiple times and the computational complexity increases linearly with the number of subbands. In addition, the noise significantly reduces the optimizing ability of the deconvolution for the spatial spectrum when the SNR is low [41]. Therefore, ISM-DCBF suffers from weak DOA estimation performance at low SNRs (as verified by the simulation and experimental results of this paper).

To achieve high-resolution LFM signal DOA estimation with a small aperture array in a low SNR hydroacoustic environment, a novel deconvolved fractional Fourier domain beamforming (DFrFB) method is proposed. The proposed method includes the following three steps. First, the LFM signals are focused in the FrFD by FrFT. Accordingly, time-domain broadband signals with low SNRs are transformed into FrFD narrowband signals with high SNRs. Second, CBF is performed to obtain a robust low-resolution spatial spectrum. Third, the high-resolution spatial spectrum is generated by a deconvolution algorithm.

Due to the coherence of LFM signals that are focused at the same time–frequency point in the FrFD, instead of deconvolving the spatial spectrum by the R-L algorithm, a monotone fast iterative shrinkage thresholding algorithm (MFISTA) [42] is introduced to realize the deconvolution of FrFB complex output. Since the FrFB complex output is not converted into beam power, the effect of cross terms between coherent sources on deconvolution can be avoided. The high-resolution DFrFB spatial spectrum is obtained by calculating the power of the deconvolution result of the FrFB complex output. The simulation and experiment show that the proposed DFrFB possesses a narrower beamwidth, lower sidelobes, and higher spatial resolution compared with FrFB and ISM-DCBF.

The remainder of this paper is organized as follows. Section 2 describes the FrFD array signal model. Section 3 introduces the FrFB and proposes the DFrFB. The convolution structure of the FrFB complex output and the shift-invariance of the complex beam pattern are also proven in Section 3. The performance of the DFrFB is demonstrated by simulations and experiments separately in Section 4 and Section 5. Section 6 is the summary of this paper.

## 2. The Fractional Fourier Domain Array Signal Model

An LFM signal with initial frequency $f_{0}$ and frequency modulation rate μ can be expressed as

(1) $$s(t)=\exp\left[j2\pi (f_{0}t+0.5\mu t^{2})\right]$$

where t∈[−T/2,T/2] and T denotes the time duration. According to the definition of FrFT in [18], the FrFT of s(t) can be expressed as

(2) $$\begin{array}{cc} S(\alpha ,u) & =F^{p}[s(t)](u)\\ \; & =\sqrt{1-j\cot\alpha }\cdot \exp(j\pi u^{2}\cot\alpha )\cdot \int_{-T/2}^{T/2}\exp\left[j(2\pi (f_{0}-u\csc\alpha )t+\pi (\mu +\cot\alpha )t^{2})\right]dt \end{array}$$

where p=2α/π is the order of FrFT (α≠nπ and n∈ℤ), $F^{p}$ denotes the FrFT operator of order p, α is the counterclockwise rotation angle (in radians) of the signal coordinate axis, and u is the peak location in the FrFT spectrum. Assume an LFM signal concentrates at $\left(\alpha_{0},u_{0}\right)$ in the FrFD, i.e., $S(\alpha_{0},u_{0})$ is the maximum of S(α,u), which means $2\pi (f_{0}-u\csc\alpha )t+\pi (\mu +\cot\alpha )t^{2}=0$ holds for any t. Thus, $\alpha_{0}$ and $u_{0}$ can be derived as

(3) $$\left\{\begin{array}{l} \alpha_{0}=-\mathrm{arccot}\mu \\ u_{0}=f_{0}\sin\alpha_{0} \end{array}\right.$$

In this case, Equation (2) can be expressed as

(4) $$\begin{array}{cc} S(\alpha_{0},u_{0}) & =F^{p_{0}}[s(t)](u_{0})\\ \; & =\sqrt{1-j\cot\alpha_{0}}\cdot \exp(j\pi u_{0}^{2}\cot\alpha_{0})\cdot T \end{array}$$

where $p_{0}$ denotes the optimal order of FrFT ($\alpha_{0}\neq n\pi$ and n∈ℤ).

Consider a uniform linear array (ULA) with M isotropic hydrophones and an LFM signal with an incidence direction of θ. The signal received at the mth hydrophone is denoted by $s(t-\tau_{m})$, where $\tau_{m}=\;(m-1)d\cos\theta /c,\;\;(m=1,2,\cdots ,M)$ is the time delay for the signal to travel from the reference hydrophone to the mth hydrophone, *d* represents the distance between adjacent hydrophones, and *c* is the speed of sound. Then, the received signal at the mth hydrophone can be expressed as

(5) $$\begin{array}{cc} x_{m}(t) & =s(t-\tau_{m})+n_{m}(t)\\ \; & =\exp(j2\pi (0.5\mu \tau_{m}^{2}-f_{0}\tau_{m}-\mu \tau_{m}t))\cdot s(t)+n_{m}(t) \end{array}$$

In Equation (5), the noise is denoted by $n_{m}(t)$, which is uncorrelated and independent of the source signal. The orientation vector of an LFM signal is time-variant and frequency-dependent, and the conventional individual frequency steering vector is inapplicable. Therefore, a frequency-independent steering vector used in the FrFD needs to be derived.

Assuming $s(t-\tau_{m})$ concentrates at $\left(\alpha_{m},u_{m}\right)$, then the FrFT of $x_{m}(t)$ can be derived as

(6) $$\begin{array}{cc} X_{m}(\alpha_{m},u_{m}) & =F^{p_{m}}[x_{m}(t)](u_{m})\\ \; & =F^{p_{m}}[s(t-\tau_{m})](u_{m})+F^{p_{m}}[n_{m}(t)](u_{m})\\ \; & =\sqrt{1-j\cot\alpha_{m}}\cdot \exp(j\pi u_{m}^{2}\cot\alpha_{m})\cdot T\cdot \exp(j\pi (-2f_{0}\tau_{m}+\mu \tau_{m}^{2}))+N_{m}(\alpha_{m},u_{m}) \end{array}$$

where $N_{m}(\alpha_{m},u_{m})=F^{p_{m}}[n_{m}(t)](u_{m})$ represents the FrFT of $n_{m}(t)$. When s(t) is delayed, the initial frequency changes from $f_{0}$ to $f_{0}-\mu \tau_{m}$, but the frequency modulation rate μ remains the same. Thus, $\alpha_{m}$ and $u_{m}$ can be represented by

(7) $$\left\{\begin{array}{l} \alpha_{m}=\alpha_{0}\\ u_{m}=u_{0}+\tau_{m}\cos\alpha_{0} \end{array}\right.$$

Substituting Equation (7) into Equation (6) and replacing $f_{0}$, μ by $\alpha_{0}$, $u_{0}$, $X_{m}(\alpha_{m},u_{m})$ can be derived as

(8) $$\begin{array}{cc} X_{m}(\alpha_{m},u_{m}) & =X_{m}(\alpha_{0},u_{0}+\tau_{m}\cos\alpha_{0})\\ \; & =A(\tau_{m})S(\alpha_{0},u_{0})+N_{m}(\alpha_{0},u_{m}) \end{array}$$

where $A(\tau_{m})=\exp[-j(2\pi \tau_{m}u_{0}\sin\alpha_{0}+\pi \tau_{m}^{2}\sin\alpha_{0}\cos\alpha_{0})]$ is the frequency-independent orientation coefficient in the FrFD. Then, $A(\tau_{m})$ can be expressed as a function of θ:

(9) $$A_{m}(\theta )=\exp(-j2\pi (m-1)d\cos\theta u_{0}\sin\alpha_{0}/c)\cdot \exp(-j\pi {\left(m-1\right)}^{2}d^{2}\cos^{2}\theta \sin\alpha_{0}\cos\alpha_{0}/c^{2})$$

## 3. Deconvolved Beamforming in the Fractional Fourier Domain

In this section, we utilize beamforming and deconvolution in the FrFD. For the simplicity of expression, one LFM target is modeled at the beginning. Then, the multitarget model is discussed when the LFM signals are concentrated on the same point.

### 3.1. Fractional Fourier Domain Delay-and-Sum Beamforming

According to the formation of Equation (9), the steering vector of an LFM signal in the FrFD can be written as

(10) $$v={\left[v_{1}(\vartheta ),v_{2}(\vartheta ),\cdots ,v_{M}(\vartheta )\right]}^{T}$$

(11) $$v_{m}(\vartheta )=\frac{1}{M}\exp(-j2\pi (m-1)d\cos\vartheta u_{0}\sin\alpha_{0}/c)\cdot \exp(-j\pi {\left(m-1\right)}^{2}d^{2}\cos^{2}\vartheta \sin\alpha_{0}\cos\alpha_{0}/c^{2})$$

where ϑ represents the beam scanning angle. Based on the aforementioned analysis, the FrFD delay-and-sum beamforming complex output is

(12) $$\begin{array}{cc} Y(\vartheta ) & =v^{H}X\\ \; & =Y_{S}(\vartheta )+Y_{N}(\vartheta ) \end{array}$$

In Equation (12), $X={\left[X_{1}(\alpha_{1},u_{1}),X_{2}(\alpha_{2},u_{2}),\cdots ,X_{M}(\alpha_{M},u_{M})\right]}^{T}$ is the matrix of the peak value in the FrFD, $Y_{N}(\vartheta )=v^{H}{\left[N_{1}(\alpha_{1},u_{1}),N_{2}(\alpha_{2},u_{2}),\cdots ,N_{M}(\alpha_{M},u_{M})\right]}^{T}$ is the beamforming complex output of noise, superscript ${\left(\cdot \right)}^{H}$ denotes the conjugate transpose, and the beamforming complex output of the LFM signal can be expressed in detail as

(13) $$\begin{array}{cc} Y_{S}(\vartheta ) & =v^{H}{\left[A_{1}(\theta )\;A_{2}(\theta )\;\cdots \;A_{M}(\theta )\right]}^{T}S(\alpha_{0},u_{0})\\ \; & =\frac{S(\alpha_{0},u_{0})}{M}\sum_{m=1}^{M}\left[\exp(-j2\pi (m-1)d(\cos\theta -\cos\vartheta )u_{0}\sin\alpha_{0}/c)\cdot \exp(-j\pi {\left(m-1\right)}^{2}d^{2}(\cos^{2}\theta -\cos^{2}\vartheta )\sin\alpha_{0}\cos\alpha_{0}/c^{2})\right] \end{array}$$

Then, the spatial spectrum of the FrFB can be given by Equation (14), and the ϑ corresponding to the peak of $P_{FrFB}(\vartheta )$ is the estimated DOA.

(14) $$P_{FrFB}(\vartheta )=|Y(\vartheta ){|}^{2}$$

For the multitarget scenario, several LFM signals will focus on different points when they have different time–frequency characteristics. In this circumstance, each target derives a corresponding spatial spectrum. The DOA is given by the unique peak of each spatial spectrum [8]. There is no need to discuss spatial resolution further, which will not be discussed in this paper. If the LFM signals from different targets possess the same time–frequency characteristics, they will focus on the same time–frequency point. Multiple LFM signals are superimposed onto each other in the FrFD. In this circumstance, the FrFB complex output can be expressed as

(15) $$Y(\vartheta )=\sum_{k=1}^{K}Y_{S,k}(\vartheta )+Y_{N}(\vartheta )$$

(16) $$Y_{S,k}=\frac{S_{k}(\alpha_{0},u_{0})}{M}\sum_{m=1}^{M}\left[\exp(-j2\pi (m-1)d(\cos\theta_{k}-\cos\vartheta )u_{0}\sin\alpha_{0}/c)\cdot \exp(-j\pi {\left(m-1\right)}^{2}d^{2}(\cos^{2}\theta_{k}-\cos^{2}\vartheta )\sin\alpha_{0}\cos\alpha_{0}/c^{2})\right]$$

In Equation (16), $Y_{S,k}$ denotes the FrFB complex output of the kth LFM signal. The spatial spectrum of FrFB for the multitarget scenario can be expressed as

(17) $$P_{FrFB}(\vartheta )={\left|\sum_{k=1}^{K}Y_{S,k}(\vartheta )+Y_{N}(\vartheta )\right|}^{2}$$

Although FrFB can effectively estimate LFM signal DOA at low SNRs, it generates a spatial spectrum with a wide mainlobe and high sidelobes because of the CBF. If two LFM signals with the same time–frequency characteristics are closely located, they will be difficult to be distinguished. A typical method for overcoming this problem is to deconvolve the spatial spectrum by the R-L algorithm. However, due to the coherence of LFM signals that focus on the same time–frequency point, this paper introduces the MFISTA algorithm to deconvolve the complex output so that the influence of cross terms between coherent sources on the spatial spectrum can be avoided.

### 3.2. Deconvolution of the Beamforming Complex Output

In this section, deconvolution is used as postprocessing for the FrFB complex output to obtain a high-resolution spatial spectrum with narrow beamwidth and low sidelobes. First, the FrFB complex output is shown to have a form of convolution, and the complex beam pattern is proved to be shift-invariant. Then, the MFISTA algorithm is introduced to realize deconvolution.

In Equation (16), the quadratic time delay term ${\left(m-1\right)}^{2}d^{2}(\cos^{2}\theta -\cos^{2}\vartheta )/c^{2}$ is proportional to the inverse of the sound speed, which is small enough to be omitted [22,43,44]. Therefore, $\exp(-j\pi {\left(m-1\right)}^{2}d^{2}(\cos^{2}\theta -\cos^{2}\vartheta )\sin\alpha_{0}\cos\alpha_{0}/c^{2})\approx 1$ and Equation (16) can be derived as

(18) $$\begin{array}{cc} Y_{S,k}(\vartheta ) & =\frac{S_{k}(\alpha_{0},u_{0})}{M}\sum_{m=1}^{M}\exp(-j2\pi (m-1)d(\cos\theta_{k}-\cos\vartheta )u_{0}\sin\alpha_{0}/c)\\ \; & =S_{k}(\alpha_{0},u_{0})\frac{\sin(\pi Md(\cos\vartheta -\cos\theta_{k})u_{0}\sin\alpha_{0}/c)}{M\sin(\pi d(\cos\vartheta -\cos\theta_{k})u_{0}\sin\alpha_{0}/c)}\exp(j(M-1)d(\cos\vartheta -\cos\theta_{k})u_{0}\sin\alpha_{0}/c) \end{array}$$

For the LFM signals, which concentrate at $\left(\alpha_{0},u_{0}\right)$ in the FrFD, the complex beam pattern can be proposed as Equation (19), according to the FrFB complex output given by Equation (18).

(19) $$Y_{P}(\vartheta |\theta )=\frac{\sin(\pi Md(\cos\vartheta -\cos\theta )u_{0}\sin\alpha_{0}/c)}{M\sin(\pi d(\cos\vartheta -\cos\theta )u_{0}\sin\alpha_{0}/c)}\exp(j(M-1)d(\cos\vartheta -\cos\theta )u_{0}\sin\alpha_{0}/c)$$

Assuming the true DOA is ψ, the source spatial distribution corresponding to a specific LFM signal is defined as follows:

(20) $$D(\theta )≜S(\alpha_{0},u_{0})\delta (\cos\theta -\cos\psi )$$

(21) δ(cosθ−cosψ)=0,   cosθ≠cosψ,   ∫δ(cosθ−cosψ)dcosθ=1

Since the complex beam pattern $Y_{P}(\vartheta |\theta )$ is a function of cosϑ−cosθ, it can be regarded as a shift-invariant function on the angle cosine, i.e., $Y_{P}(\cos\vartheta |\cos\theta )=Y_{P}(\cos\vartheta -\cos\theta )$. Therefore, $Y_{P}(\cos\vartheta |\cos\theta )$ is shift-invariant. Hence, the complex output of LFM signals can be expressed in a convolution formation as

(22) $$Y_{S}(\cos\vartheta )=\int Y_{P}(\cos\vartheta -\cos\theta )D(\cos\theta )d\cos\theta$$

Therefore, the deconvolution algorithm can be utilized to restore D(cosθ), with the knowledge of $Y_{S}(\cos\vartheta )$ and $Y_{P}(\cos\vartheta -\cos\theta )$. Considering that both $Y_{S}(\cos\vartheta )$ and $Y_{P}(\cos\vartheta -\cos\theta )$ are complex values, the typical R-L algorithm is no longer applicable. The deconvolution of Equation (22) is converted into a sparse-constrained optimization problem, as shown in Equation (23).

(23) $$\underset{\hat{D}\in {\mathbb{C}}^{G}}{\arg\min}\frac{1}{2}{\left\|Y_{S}-Y_{P}\hat{D}\right\|}_{2}^{2}+\lambda {\left\|\hat{D}\right\|}_{1}$$

(24) $$Y_{P}={\left[Y_{P}(\theta_{ri}|\theta_{ci})\right]}_{G\times G}$$

In Equation (23), $Y_{S},\hat{D}\in {\mathbb{C}}^{G}$ are the FrFB output vector and the target signal space distribution vector, respectively. $Y_{P}\in {\mathbb{C}}^{G\times G}$ is the complex beam pattern matrix, λ∈ℝ is the regularization coefficient, and G is the number of discrete angles. In Equation (24), $ri,ci\in [1,G]\subset {\mathbb{Z}}^{+}$ represent the indices of the row and column in the matrix, respectively, and $\left[\theta_{1},\theta_{2}\cdots ,\theta_{G}\right]$ represents the set of discrete angles. The solution to Equation (23) can be obtained by the MFISTA Algorithm 1, which is given as follows.
**Algorithm 1:** MFISTA algorithm.**Input:** FrFB complex output $Y_{S}$, complex beam pattern $Y_{P}$, regularization coefficient λ, iteration times I, and initial value ${\hat{D}}_{0}$;**Output:** the estimation of source signal distribution ${\hat{D}}_{I}$;
1. **set**
$l_{1}=1$$\;\mathrm{and}\;y_{1}={\hat{D}}_{0}$;
2. **for**
i=1 to I
3.   $z_{i}=S_{\lambda /L}(\frac{1}{L}Y_{P}^{H}(Y_{S}-Y_{P}y_{i})+y_{i})$;4.   
${\hat{D}}_{i}=\underset{\hat{D}\in \{z_{i},{\hat{D}}_{i-1}\}}{\arg\min}\frac{1}{2}{\left\|Y_{S}-Y_{P}\hat{D}\right\|}_{2}^{2}+\lambda {\left\|\hat{D}\right\|}_{1}$;
5.   
$l_{i+1}=(1+\sqrt{1+4l_{i}^{2}})/2$;
6.   $y_{i+1}={\hat{D}}_{i}+\frac{l_{i}-1}{l_{i+1}}({\hat{D}}_{i}-{\hat{D}}_{i-1})+\frac{l_{i}}{l_{i+1}}(z_{i}-{\hat{D}}_{i})$;
7. **end**

In Step 3 of Algorithm 1, L denotes the step size, and the shrinkage-thresholding function $S_{\lambda /L}(x)$ is defined for arbitrary $x={\left[x_{1},x_{2},\cdots ,x_{G}\right]}^{T}\in {\mathbb{C}}^{G}$ by

(25) $$S_{\lambda /L}(x_{g})=\left\{\begin{array}{ll} 0 & \left|x_{g}\right|<\frac{\lambda }{L}\\ x_{g}-\frac{\lambda x_{g}}{L\left|x_{g}\right|} & \left|x_{g}\right|\geq \frac{\lambda }{L} \end{array}\right.$$

After obtaining the estimation ${\hat{D}}_{I}$ using Algorithm 1, the DFrFB spatial spectrum can be expressed as

(26) $$P_{DFrFB}={\left|{\hat{D}}_{I}\right|}^{2}$$

**Figure 1:**
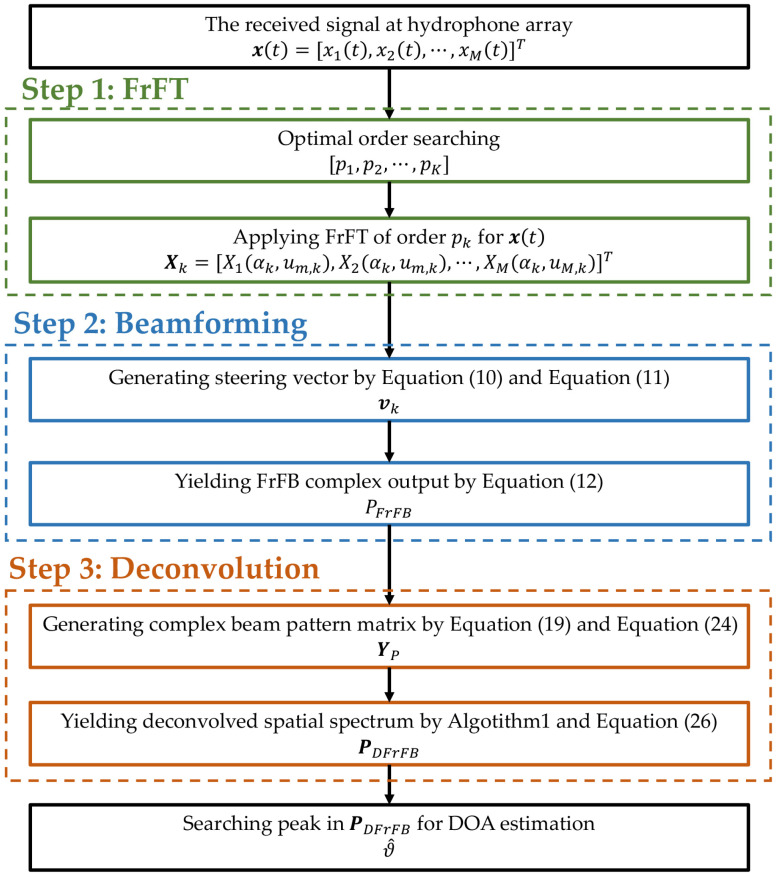
Flowchart of the proposed DFrFB method.

The flowchart of the proposed DFrFB is shown in Figure 1. A coarse-to-fine searching strategy [24] is utilized to obtain the optimal order of the FrFT. The discrete FrFT is implemented using the algorithm proposed in [16]. In the MFISTA deconvolution algorithm, the step size is set to the maximum eigenvalue of the covariance matrix of $Y_{P}$, and the regularization coefficient λ is set to 0.5.

## 4. Simulation Results

In this section, the performance of DFrFB is verified by five simulations. The same simulations are performed for FrFB and ISM-DCBF for reference. The root mean square error (RMSE) of the DOA estimation and resolution probability (RP) are calculated to evaluate the spatial resolution. The beamwidth and maximum sidelobe level are recorded to evaluate the optimization of the space spectrum. When the estimated DOAs $\hat{\theta_{1}},\hat{\theta_{2}}$ and the real DOAs $\theta_{1},\theta_{2}$ satisfy Equation (27), two targets are considered to be successfully discriminated. Assuming $N_{total}$ times Monte Carlo experiments are performed and $N_{success}$ times experiments satisfy Equation (27), the RP is defined as $N_{success}/N_{total}$. The RMSE is calculated only when the RP is 1. The beamwidth is also referred to as the half-power beamwidth, i.e., the width of the mainlobe at −3 dB.

(27) $$\left|\hat{\theta_{1}}-\theta_{1}\right|+\left|\hat{\theta_{2}}-\theta_{2}\right|<\left|\theta_{1}-\theta_{2}\right|$$

The LFM signals emitted by different targets are identical, and the detailed parameters are given in Table 1. The noise signal is Gaussian white noise, and the noise signal received on each hydrophone is independently identically distributed and unrelated to the target signals. The SNR is defined by Equation (28), where $P_{S,k}$ denotes the power of the kth target signal and $P_{N}$ denotes the power of the noise.

(28) $$SNR=10\log_{10}(\sum_{k=1}^{K}P_{S,k}/P_{N})$$

All time-domain snapshots are processed together by fast Fourier transform (FFT) or FrFT in the corresponding methods. In ISM-DCBF, the received signal is divided into four subbands, and MFISTA is also used as the deconvolution algorithm. Unless stated otherwise, the number of deconvolution iterations is 5000 in ISM-DCBF and DFrFB. The number of iterations of ISM-DCBF refers to that of one subband, and each subband has the same number of iterations. A ULA with 10 hydrophones is used as the received array, whose element spacing is 0.1 m. The sound speed is 1500 m/s. The number of Monte Carlo simulations in Section 4.2, Section 4.3, Section 4.4 and Section 4.5 is 100. All simulations in this paper are conducted by MATLAB R2020a.

### 4.1. Spatial Spectrum

**Figure 2:**
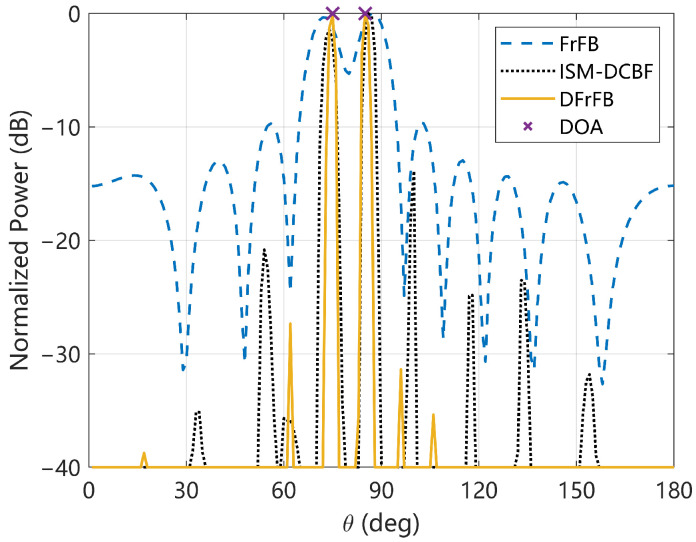
Spatial spectra of the simulation.

The spatial spectra of the FrFB, ISM-DCBF, and DFrFB at 0 dB are given in Figure 2. The cross symbol represents the real DOA of the target, which is 75° and 85°. The estimated DOAs, beamwidth, and sidelobe levels are given in Table 2. As shown in Figure 2, the three methods are all successful in identifying two targets. The estimation error of DFrFB is the smallest, ISM-DCBF is the second smallest, and FrFB is the largest. By deconvolving the complex output of the FrFB, the beamwidth is reduced by 75%, and the sidelobe level is reduced by 17.7 dB, resulting in a much cleaner DFrFB spatial spectrum.

Since the energy of the LFM signal is highly concentrated in the FrFD whereas the noise is not [24], noise shows less influence on DFrFB. In contrast, noise shows a large influence on each ISM-DCBF subband signal. Thus, the number and intensity of DFrFB sidelobes are smaller than those of ISM-DCBF. It is demonstrated that DFrFB can effectively improve the spatial spectrum of FrFB and has better performance than ISM-DCBF, which is based on frequency-domain signal processing.

### 4.2. The Number of Deconvolution Iterations

**Figure 3:**
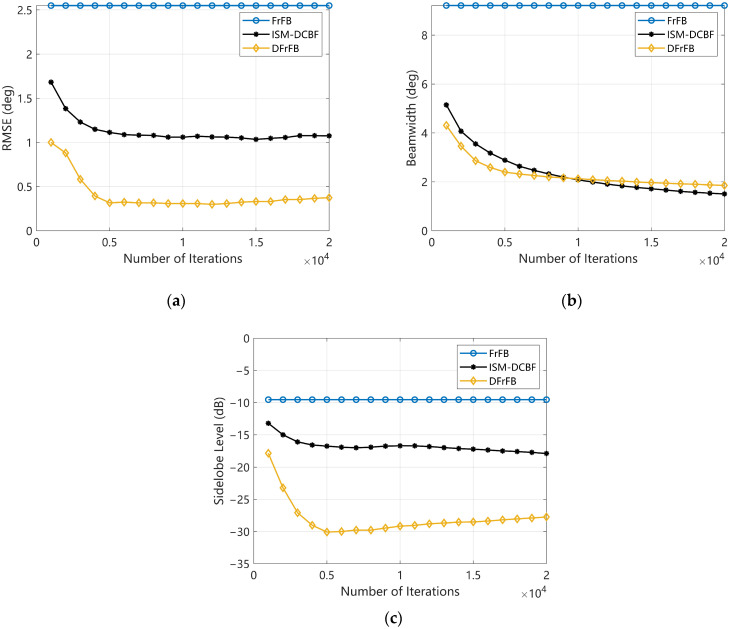
The performances of FrFB, ISM-DCBF, and DFrFB versus the number of iterations. (**a**) The RMSE; (**b**) the beamwidth; (**c**) the sidelobe level.

The influence of the number of iterations in the MFISTA algorithm on DFrFB and ISM-DCBF is discussed in this section. The source direction and SNR are consistent with Section 4.1. The RMSE, beamwidth, and sidelobe level with the number of iterations are given in Figure 3a, Figure 3b and Figure 3c, respectively. Note that FrFB requires no deconvolution, and its data are independent of the number of iterations but still plotted in Figure 3 as a reference. The RP of all three methods is 1 regardless of the number of iterations, so the RP figure with the number of iterations is not given.

All the indicators of DFrFB and ISM-DCBF rapidly improve with the increase in iterations when the number is less than 5000. DFrFB performs better than ISM-DCBF for the same number of iterations. However, the effect of noise is magnified by the deconvolution algorithm when the number of iterations exceeds 5000. Only beamwidth can be reduced further. For DFrFB, the RMSE and sidelobe level no longer improve with increasing iterations but deteriorate slightly. For ISM-DCBF, the optimization of RMSE and sidelobe level is minimal and almost stagnant. Figure 3 shows that the DFrFB performs better than ISM-DCBF when the number of iterations is appropriate. However, the number of iterations should not be too large; otherwise, the DFrFB performance will deteriorate in turn.

### 4.3. SNR

**Figure 4:**
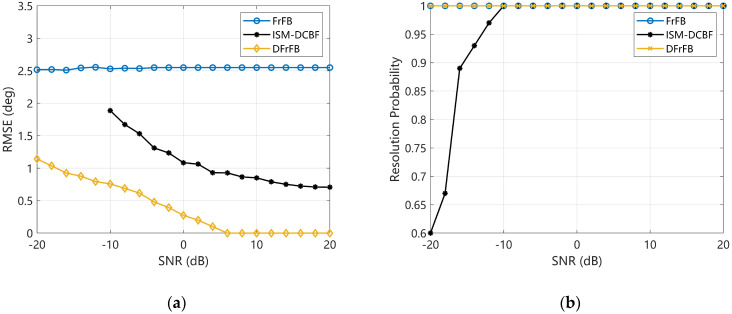
The performances of FrFB, ISM-DCBF, and DFrFB versus the SNR of the received signal. (**a**) The RMSE; (**b**) the RP.

This section investigates the effect of noise on the spatial resolution with the SNR ranging from −20 dB to 20 dB. The real DOAs of the two targets remain 75° and 85°. As shown in Figure 4a, the FrFB is insensitive to noise, and its RMSE fluctuates slightly with the SNR. However, the estimation error is large regardless of the SNR. The RMSEs of DFrFB and ISM-DCBF decrease with increasing SNR. The DFrFB accuracy is better than that of ISM-DCBF, showing better noise resistance. In Figure 4b, benefiting from the FrFT energy-focusing property, the RP of FrFB and DFrFB remains 1 even if the SNR is as low as −20 dB. In contrast, ISM-DCBF reaches an RP of 1 only when the SNR is higher than −12 dB. The DFrFB based on FrFD signal processing demonstrated better spatial resolution at low SNRs compared with ISM-DCBF based on frequency-domain signal processing.

### 4.4. The Number of Hydrophones

**Figure 5:**
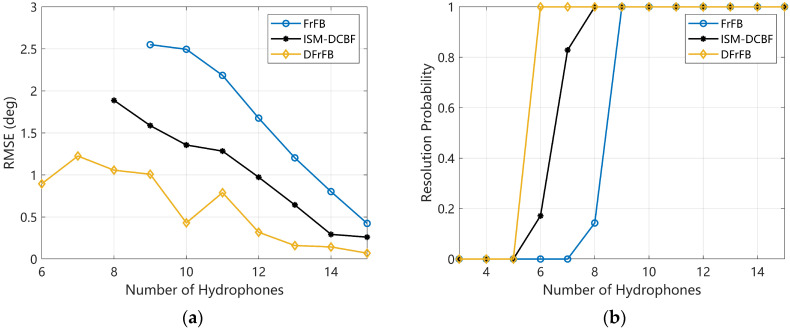
The performances of FrFB, ISM-DCBF, and DFrFB versus the number of hydrophones. (**a**) The RMSE; (**b**) the RP.

In this section, the effect of the number of hydrophones on the spatial resolution of the DFrFB is studied. The SNR is 0 dB, and the real DOAs of the two targets are the same as in Section 4.3. The RMSE and RP for the number of hydrophones varying from 3 to 15 are shown in Figure 5. DFrFB obtains the smallest DOA estimation error in Figure 5a. As shown in Figure 5b, to reach an RP of 1, DFrFB needs at least six hydrophones, while ISM-DCBF needs eight and FrFB needs nine. DFrFB can realize more accurate DOA estimations with fewer hydrophones. It is shown that DFrFB can obtain a high spatial resolution with a small aperture array.

Note that the RMSE of DFrFB does not decrease steadily with the increase in hydrophones. The ideal peak location $u_{m}$ given by Equation (7) is most likely not an integer. However, the peak location selected in practice is an integer due to the received signal being discrete in the time domain. This leads to a roundoff error of $u_{m}$ and a model mismatch when deconvolution is applied. When the number of hydrophones changes, the roundoff error also changes accordingly. Hence, the roundoff error leads to obvious fluctuations in the RMSE of DFrFB. Nevertheless, DFrFB still obtains the most accurate DOAs.

### 4.5. Angular Interval

**Figure 6:**
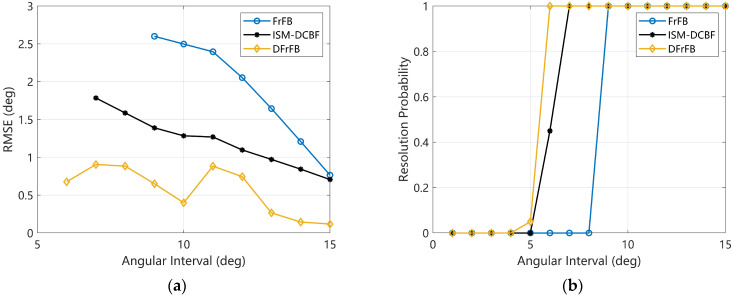
The performances of FrFB, ISM-DCBF, and DFrFB versus different angular intervals. (**a**) The RMSE; (**b**) the resolution probability.

Various angular intervals are simulated in this section to verify the adjacent target discrimination capability of the proposed DFrFB. One target is fixed at 85°, and another target moves from 84° to 70° so that the angular interval gradually changes from 1° to 15°. The number of hydrophones in the ULA is 10. Figure 6a,b show the RMSE and RP with various angular intervals when the SNR is 0 dB. The reason for the fluctuations in the RMSE of DFrFB is similar to that in Section 4.4, which is not redescribed here.

In Figure 6a, despite the apparent fluctuations, DFrFB obtains the most accurate DOA estimations regardless of the angular interval. The minimum discriminable angular interval of the DFrFB is 6°, while that of the ISM-DCBF and FrFB is 7° and 9°, respectively. It is demonstrated that DFrFB possesses the highest adjacent target discrimination capability.

## 5. Experimental Results

The DFrFB performance is verified by an experiment conducted at Jingye Lake, Tianjin, China. Jingye Lake is broadly rectangular in shape, approximately 126 m long, 80 m wide, and 3 m deep on average. The average sound speed is 1476 m/s, measured by a Valeport miniSVP. In the experiment, the target source continuously emits multiple frames of the same LFM signal, and the time interval between two adjacent frames is 0.25 s, so that no interference between different frames can be guaranteed. The detailed LFM signal parameters can be seen in Table 1. The real DOAs of the two targets are 92.2° and 116.5°, measured by the $NTS-382R_{6}$ total station. The receiving ULA consists of eight Brüel & Kjær 8104 hydrophones with an element spacing of 0.8 m. The receiving array is deployed horizontally. Both the receiving and transmitting ends are deployed at 1 m depth to ensure that the target and the array are in the same plane. The received signal is processed by a digital filter with a passband of 7~8 kHz, and the filtered SNR is approximately 4.1 dB. Only the received signal containing the LFM signal is beamformed, and the pure noise received signal is not involved in the calculation. The deconvolution iterations of DFrFB and ISM-DCBF are still 5000.

**Figure 7:**
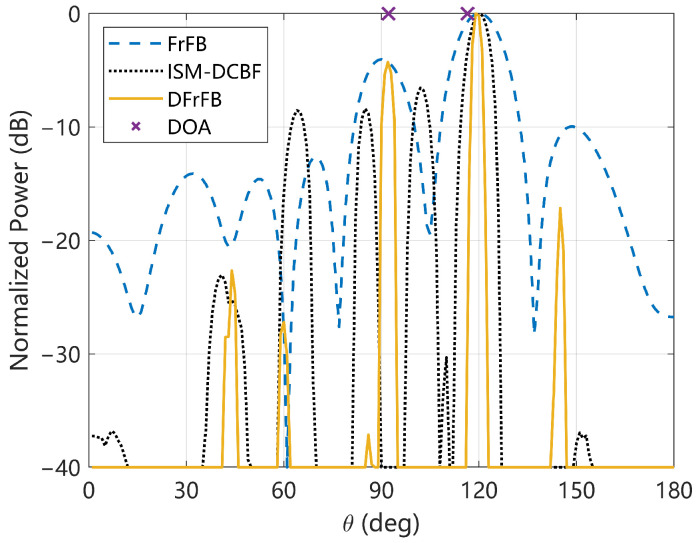
The spatial spectrum of a one-frame LFM signal received in the experiment.

The spatial spectrum of a one-frame LFM signal is given in Figure 7, where the cross symbols represent the real target DOAs. All three methods yield relatively accurate DOAs for the target located at 116.5°. However, the sidelobe of ISM-DCBF is so significant that it affects the estimation of another target. The sidelobes of DFrFB, in contrast, show no significant effect on detecting the two targets. Due to the complexity of the hydroacoustic channel in the lake and the inaccuracy of the hydrophone installation positions, a slight model mismatch occurs and the spatial spectral optimization capability of both the DFrFB and ISM-DCBF is reduced.

**Figure 8:**
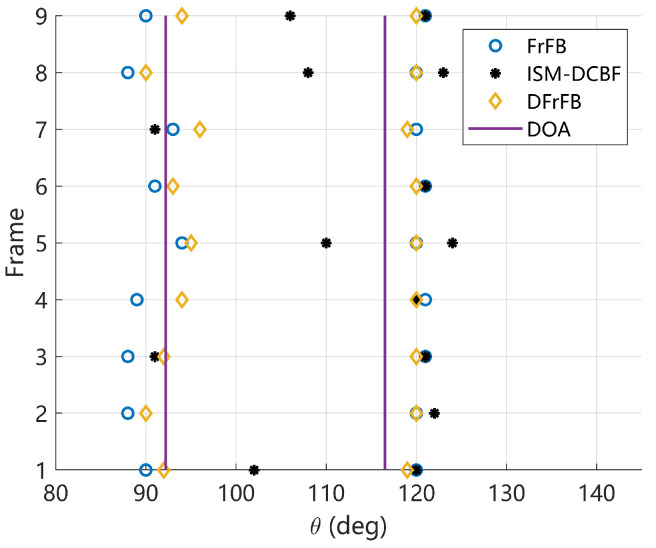
The estimated DOAs of nine frames of LFM signals received in the experiment.

The DOA estimations of nine consecutive frames are given in Figure 8, where the vertical lines represent the real DOAs. The average RMSE, beamwidth, and sidelobe level are listed in Table 3. DFrFB effectively optimizes the spatial spectrum of FrFB under experimental conditions. Compared with FrFB, DFrFB reduces the beamwidth by 77% and the sidelobe by 6.0 dB, resulting in more accurate DOA estimations. Compared with ISM-DCBF, DFrFB yields more stable and accurate DOA estimations, as well as a cleaner spatial spectrum. It is demonstrated that the proposed DFrFB possesses the best performance among the three methods.

## 6. Summary

The proposed DFrFB can achieve high-resolution LFM signal DOA estimation by a small aperture array in a low SNR hydroacoustic environment. In this paper, the FrFD array signal model is established. The FrFB complex output is proven to be the convolution of the shift-invariant complex beam pattern with the source signal spatial distribution. The MFISTA deconvolution algorithm is introduced to solve the deconvolution of complex values. Additionally, a robust high-resolution spatial spectrum is obtained. The results of the simulation and experiment show that the proposed DFrFB can effectively improve the FrFB performance and achieve a high-resolution DOA estimation of multiple LFM signals. In addition, DFrFB possesses better performance compared with directly performing DCBF for the LFM signal frequency-domain subbands.

## Figures and Tables

**Table 1:** Parameters of LFM signal.

Parameter	Value
beamwidth	1 kHz
center frequency	7.5 kHz
frequency modulation rate	20 kHz
time duration	0.05 s
sampling rate	62.5 kHz

**Table 2:** The performance of the simulation.

Indicators	FrFB	ISM-DCBF	DFrFB
DOA (deg)	72, 87	74, 86	75, 85
beamwidth (deg)	9.3	3.6	2.3
sidelobe level (deg)	−9.6	−13.9	−27.3

**Table 3:** The performance of the experiment.

Indicators	FrFB	ISM-DCBF	DFrFB
RMSE (deg)	3.5	15.1	2.8
beamwidth (deg)	14.5	5.7	3.3
sidelobe level (dB)	−10.1	−7.3	−16.1

## Data Availability

Data are available on request.
